# Case report: Giant pituitary neuroendocrine tumor presented along with acute visual loss due to pituitary apoplexy after receiving COVID-19 vaccination

**DOI:** 10.3389/fsurg.2023.1220098

**Published:** 2023-07-27

**Authors:** Haku Tanaka, Fumihiko Nishimura, Kenta Nakase, Shohei Yokoyama, Ichiro Nakagawa, Shuichi Yamada, Kentaro Tamura, Ryosuke Matsuda, Yasuhiro Takeshima, Masashi Kotsugi, Young-Soo Park, Hiroyuki Nakase

**Affiliations:** Department of Neurosurgery, Nara Medical University, Kashihara, Japan

**Keywords:** covid vaccination, pituitary apoplexy, endonasal endoscopic surgery abbreviations COVID-19, giant pituitary neuroendocrine tumor, coronavirus disease 2019, MRI magnetic resonance imaging

## Abstract

**Objective:**

A case of giant pituitary neuroendocrine tumor presented along with acute visual loss due to pituitary apoplexy after receiving a COVID-19 vaccination is reported.

**Case presentation:**

A 45-year-old man was referred for a giant pituitary tumor with bitemporal hemianopsia. A surgical procedure was planned and then delayed due to the COVID-19 outbreak in Japan, with a Pfizer/BioNTech vaccine administered while awaiting surgery. Three days after the second COVID-19 vaccination the patient noted a progressively worsening headache that caused pituitary apoplexy and then a decrease in vision. Emergency surgery was thus performed.

**Conclusion:**

Pituitary apoplexy is a rare and life-threatening complication that may occur after undergoing a COVID-19 vaccination.

## Introduction

Severe acute respiratory syndrome coronavirus 2 (SARS-CoV-2) is a non-segmented positive-sense single-stranded ribonucleic acid (RNA) beta coronavirus that first appeared in Wuhan, China (1). A SARS-CoV-2 infection causes coronavirus disease 2019 (COVID-19), which became a global pandemic and public health crisis (2). For overcoming risk factors associated with this pandemic, vaccination medications have been developed as a safe and effective means to provide protection and reduce disease spread, which has been confirmed in large clinical trials (3). Common adverse events related to vaccine administration include mild-to-moderate tenderness at the injection site, fever, fatigue, body aches, and headaches. However, no association of COVID-19 vaccination with pituitary apoplexy has been established, as few cases have been reported (4–6). Herein, we present the first known case of pituitary apoplexy that developed soon after receiving a Pfizer/BioNTech (BNT162b) COVID-19 vaccination.

## Case description

A 45-year-old male was referred to our hospital with the chief complaints of bitemporal hemianopsia and vision loss (visual acuity, left: 0.04, right: 0.03) (Supplementary Figure S1). Brain magnetic resonance imaging (MRI) revealed a giant pituitary tumor which was compressing to optic apparatus and bilateral basal ganglia (Supplementary Figure S2), while endocrine findings obtained at that time showed no evidence of a functional pituitary neuroendocrine tumor (Supplementary Table S1). Moreover, the level of cortisol was very low, suggesting hypoadrenalism, although thyroid function was still within the reference range (Supplementary Table S1). The levels of testosterone, growth hormone (GH) and insulin like growth factor 1 (IGF-1) were also low, suggesting hypogonadism and GH deficiency (Supplementary Table S1). On the other hand, the level of prolactin (PRL) was elevated, suggesting stalk effect by a giant pituitary tumor (Supplementary Table S1).

A surgical procedure was planned as soon as possible, however, at this point, due to the COVID-19 outbreak in Japan, a second Pfizer/BioNTech vaccine was already scheduled and administered 1 month after a first visit to our department while awaiting the operation. As a result, the operation was planned 2–3 weeks after the second COVID-19 vaccination.

**Figure 1:**
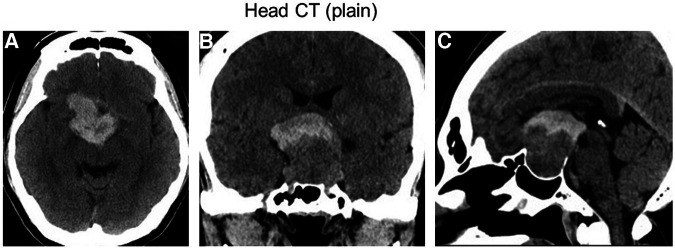
An emergency CT examination performed 3 days after COVID-19 vaccination showed a hyperdense suprasellar mass lesion in the upper part of the known pituitary tumor, suggesting pituitary apoplexy. (**A**) Axial section. (**B**) Coronal section. (**C**) Sagittal section.

Three days after the second COVID-19 vaccine injection, the patient was presented to our emergency department because of a progressively worsening headache and vomiting. Emergency computed tomography (CT) results showed a hyperdense suprasellar mass lesion in the upper part of the known pituitary tumor (Figure 1). Furthermore, vision was decreased to finger counting at 30 cm. Because of partial hypopituitarism as shown on Supplementary Table S1, we assumed that the patient with pituitary apoplexy had adrenal insufficiency. Hydrocortisone (dose with 100 mg), therefore, was administered intravenously on admission, and then, 200 mg of hydrocortisone per day was continued to infuse during and after the surgery to prevent from adrenal crisis as replacement therapy.

Although laboratory data on the coagulation system showed that prothrombin time (PT) was slightly prolonged, there was no apparent evidence of vaccine-induced thrombotic thrombocytopenia (Supplementary Table S2).

**Figure 2:**
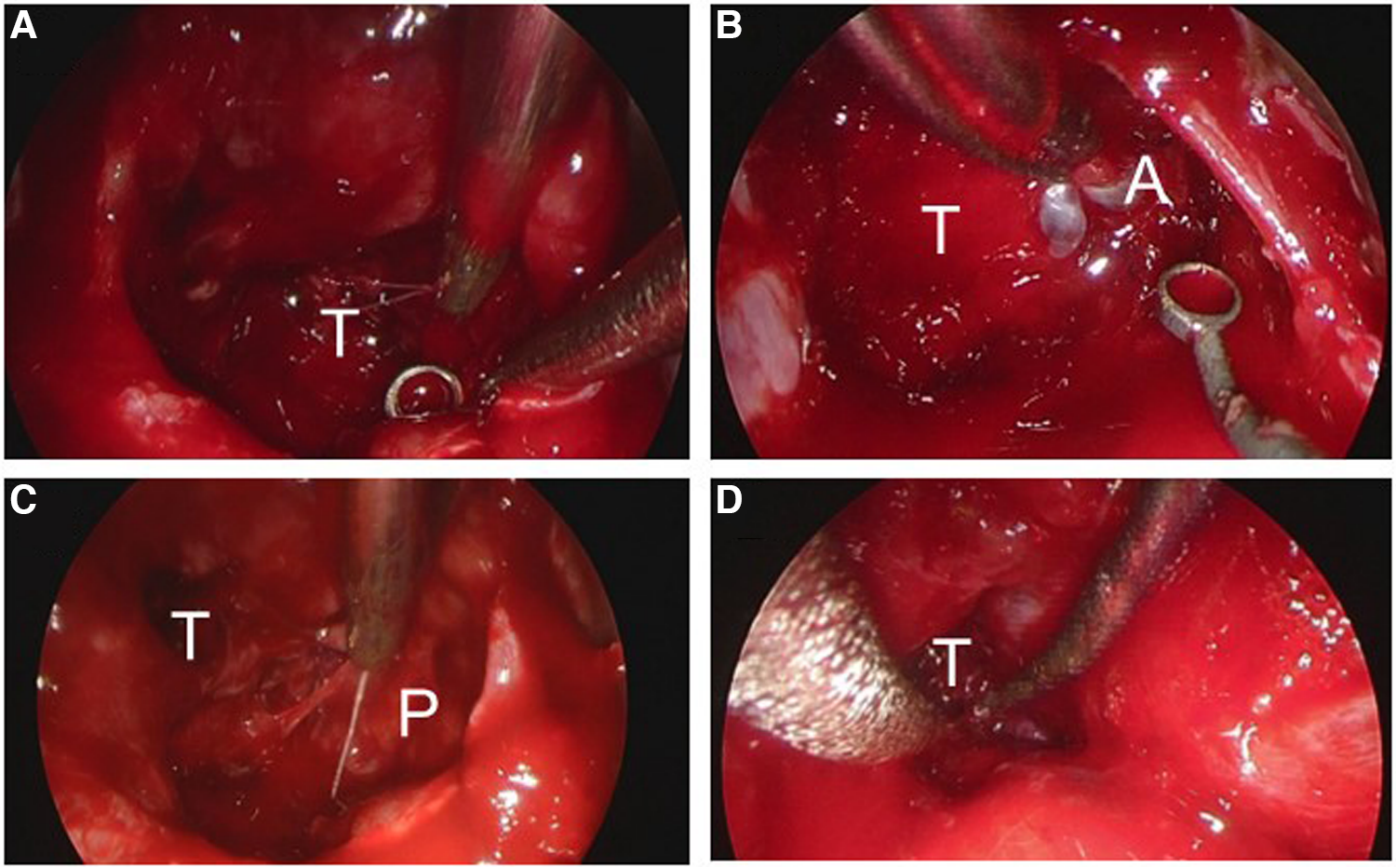
Images obtained during endoscopic endonasal surgery. (**A**) A tumor with hematoma was debulked in a piecemeal manner. (**B**) The arachnoid membrane on the left side of the diaphragm was exposed. (**C**) Compression of the pituitary gland was observed on the left side with the patient in a superior position. (**D**) The hard tumor was intentionally left in place. T, tumor; A, arachnoid; P, pituitary gland.

**Figure 3:**
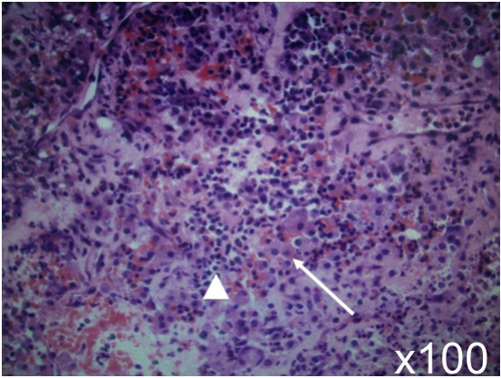
Histopathologic analysis led to a diagnosis of pituitary neuroendocrine tumor. The findings showed tumor cells with eosinophilic cytoplasm (white arrow) invaded by lymphocytes (white arrowhead) and red blood cells suggesting pituitary apoplexy. Hematoxylin and eosin staining; original magnification x100.

An emergency endoscopic endonasal transsphenoidal surgical procedure was performed after confirming negative COVID-19 polymerase chain reaction (PCR) test results. Intraoperative findings revealed an intrasellar mass lesion that formed a relatively soft tumor similar to an ordinary pituitary neuroendocrine tumor (Figure 2A). Following resection of the intrasellar mass, the arachnoid membrane on the left side of the diaphragm was exposed (Figure 2B), which revealed compression of the pituitary gland on the superior left side (Figure 2C). Furthermore, on the superior right side a hemorrhagic necrotic hard tumor was observed. The hard tumor was intentionally left, as removal was considered to be dangerous and could potentially cause damage to the neurovascular structure (Figure 2D). Based on histopathology results, the diagnosis was pituitary neuroendocrine tumor (Figure 3). The findings showed tumor cells with eosinophilic cytoplasm (white arrow) invaded by lymphocytes (white arrowhead) and red blood cells suggesting pituitary apoplexy (Figure 3).

**Figure 4:**
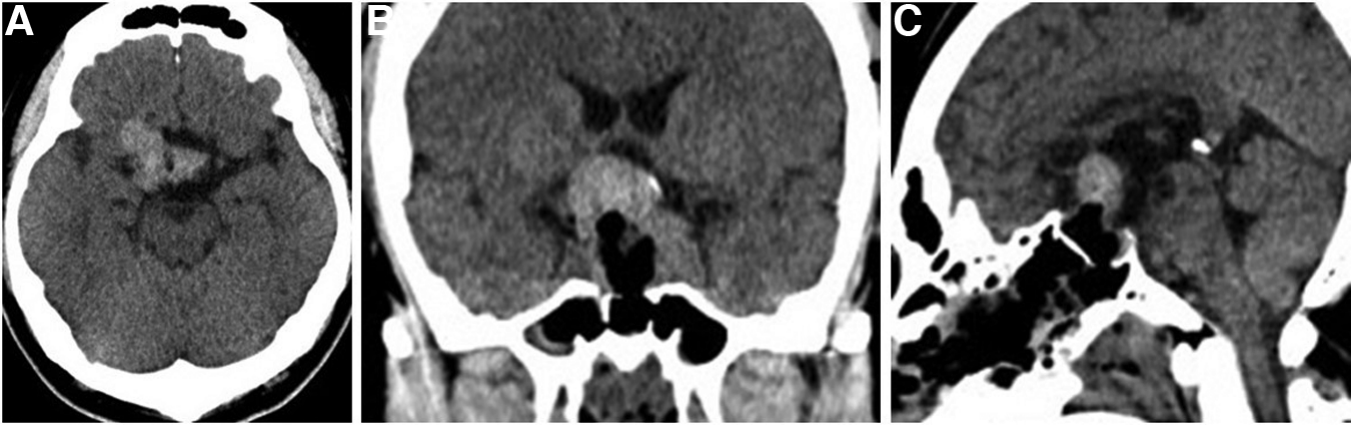
Postoperative head CT showed the residual tumor with hematoma in the suprasellar region. (**A**) Axial section. (**B**) Coronal section. (**C**) Sagittal section.

Following surgery, the headache was improved and improvement of visual acuity in the left eye to 0.7 was noted. However, as compared to visual function status findings before apoplexy development, visual acuity and visual field impairment in the right eye had worsened (visual acuity, right: 0.01) (Supplementary Figure S3). The patient developed diabetes insipidus (DI) with thirst and hypotonic polyuria during the immediate postoperative period, which was controlled with oral desmopressin. Postoperative CT findings showed the residual tumor with hematoma in the suprasellar region (Figure 4). Dynamic endocrine test results after discharge indicated panhypopituitarism, thus various hormone replacements were administered. Five months later, brain MRI results showed tumor shrinkage because of natural hematoma absorption and decompression of the optic apparatus (Supplementary Figure S4). Eye examinations are continuing as part of the clinical course, with no additional radiation therapy performed at the time of writing.

## Discussion

Pituitary apoplexy is a rare neurological and endocrine emergency disease, with recent reports showing an annual prevalence of 6.2 and incidence of 0.17 per 100,000 in the general population (7–9). However, results of two meta-analyses suggest that the risk of pituitary apoplexy in patients known to have a nonfunctional pituitary neuroendocrine tumor and treated conservatively may range from 0.2 to 0.6 events per 100 person-years (10, 11). While the mechanism of pituitary apoplexy is unclear, it is generally considered more likely to occur in patients with a giant pituitary adenoma, with cardiac surgery, major surgery, pregnancy, cerebral angiography, and dynamic endocrine testing considered to be precipitating factors (12–14). The most commonly mentioned speculation is that infarction and hemorrhage occur due to the peculiarities and fragility of the complex vascular structure of a normal pituitary gland and pituitary tumor, along with an imbalance of necessary blood supply (15). There is another differential diagnosis of pituitary apoplexy which is caused by anabolic androgenic steroids abuse resulting in abnormally high testosterone levels (16).

Recently, reports of pituitary apoplexy in COVID-19 infected patients have increased, with the virus identified as a possible precipitating risk factor for pituitary apoplexy (17, 18). COVID-19 binds to the angiotensin-converting enzyme 2 (ACE2) receptor, which induces down-regulation of the renin angiotensin system (RAS), resulting in multiple organ failure, increased sympathetic nerve activity, disruption of blood pressure autoregulation, and increased production of vasoconstrictor inflammatory cytokines (19). Moreover, ACE2 receptors are also expressed in pituitary tissue, thus COVID-19 may be more likely to damage pituitary tissue because it enters via the olfactory nerve (20, 21). For these reasons, COVID-19 is considered as a plausible risk factor for pituitary apoplexy. However, since endonasal surgery poses a risk of COVID-19 infection to medical staff, a preoperative vaccination may be advisable.

Several reports of pituitary apoplexy in patients that have been vaccinated for COVID-19 have been presented (4–6). As mentioned in the study reported by Aliberti et al, the possibility of occult COVID-19 infection cannot be excluded. Even after confirming negative COVID-19 polymerase chain reaction (PCR) test results in this case, there was a possibility of occult COVID-19 infection although we did not confirm the absence of SARS-CoV-2 nuclear protein in the pathologic specimen.

Piñar-Gutiérrez et al. reported a case of pituitary apoplexy after vaccination with ChadOx1-S, in which the patient came to the clinic with a worsening headache at 5 days after the injection (5). Head MRI was performed and the findings showed hemorrhagic changes in the pituitary gland. Normal results were obtained with campimetry and pituitary analysis, and conservative therapy led to a resolution of symptoms. The authors considered possible vaccine-induced thrombotic thrombocytopenia (VITT), though thrombocytopenia could not be confirmed because of no available hemogram findings.

Although there was a report of pituitary apoplexy after COVID-19 mRNA vaccine (Moderna) (4), no known case of pituitary apoplexy after another type of mRNA vaccination (Pfizer-BNT162b) has been reported, while there are also no data available showing that a BNT vaccine predisposes the recipient to a stroke. However, cases of stroke after vaccination have been noted, while the possibility that occurrence of myocarditis, vasculitis, or Guillain-Barré is more likely has also been noted. The related mechanism in the present case is unknown and may be unrelated to the vaccine, though it is possible that an immune response to the vaccine or stress caused by a side-effect of the vaccine may have had an influence on pituitary apoplexy development. With this awful experience of pituitary apoplexy after vaccination, we would recommend surgical procedure for especially giant PitNET before vaccination in case of apoplexy.

Pituitary apoplexy symptoms and side-effects of BNT162b are similar, with the latter reported to include a severe headache and high fever (22). On the other hand, pituitary apoplexy is known to cause a headache in 80%–90% of affected patients and a fever in 16% (23). Therefore, mild pituitary apoplexy after a COVID-19 vaccination may be an underdiagnosed condition.

With regard to definition and management of giant pituitary neuroendocrine tumors (GPitNETs) according to EANS, the European Association of Neurosurgical.

Societies, GPitNETs are defined with a maximal diameter >40 mm on cerebral MRI and the endoscopic endonasal approach is recommended as the first option (24, 25). Our case was GPitNET with a maximal diameter of 44.2 mm, and this case undertook the endoscopic endonasal surgery to decompress the optic apparatus successfully.

## Conclusion

Although the precise mechanism of pituitary apoplexy development following receipt of an mRNA vaccine is unknown, there is a possibility that an immune response to the vaccine or stress caused by a side-effect of vaccine may have had an influence on pituitary apoplexy development. Further results of additional cases need to be accumulated. Moreover, symptoms related to pituitary apoplexy are similar to vaccine side-effects, thus caution is advised, especially for patients with a medical history of pituitary tumor.

## Data Availability

The original contributions presented in the study are included in the article/**Supplementary Materials**, further inquiries can be directed to the corresponding author.

## Supplementary material

Supplementary Figure S1

An ophthalmologic evaluation using Goldmann perimetry indicated bitemporal hemianopsia. Visual acuity was examined with a Landolt ring, which revealed loss of visual acuity.

Supplementary Figure S2

Brain MRI following gadolinium (Gd) administration showed a well-enhanced intra- and suprasellar giant mass lesion. (A) Axial section. (B) Coronal section. (C) Sagittal section.

Supplementary Figure S3

An ophthalmologic evaluation with Goldmann perimetry one week after surgery revealed bitemporal hemianopsia. Visual acuity was determined with a Landolt ring, with only that in the left eye found to be improved.

Supplementary Figure S4

Five months after surgery, brain MRI findings showed tumor shrinkage because of natural hematoma absorption and decompression of the optic apparatus. (A) Axial section. (B) Coronal section. (C) Sagittal section.

Supplementary Table S1

Hormone findings in blood. Downward arrows indicate below minimal range and upward arrows above maximal range. TSH, thyroid stimulating hormone; LH, luteinizing hormone; FSH, follicle-stimulating hormone; PRL, prolactin; ACTH, adrenocorticotropic hormone; GH, growth hormone; IGF-1, insulin-like growth factor-1; AVP, arginine vasopressin.

Supplementary Table S2

Coagulation system findings in blood. Upward arrow indicates above maximal range. WBC, white blood cell; RBC, red blood cell; Hb, hemoglobin; Ht, hematocrit; PT, prothrombin time; APTT, activated partial thromboplastin time.
