# Supplementary material for: Case report: Giant pituitary neuroendocrine tumor presented along with acute visual loss due to pituitary apoplexy after receiving COVID-19 vaccination
Source: Front Surg. 2023 Jul 27;10:1220098. doi: 10.3389/fsurg.2023.1220098 (PMC10413133; doi:10.3389/fsurg.2023.1220098)

Suppl. Figure 1.

Left eye field

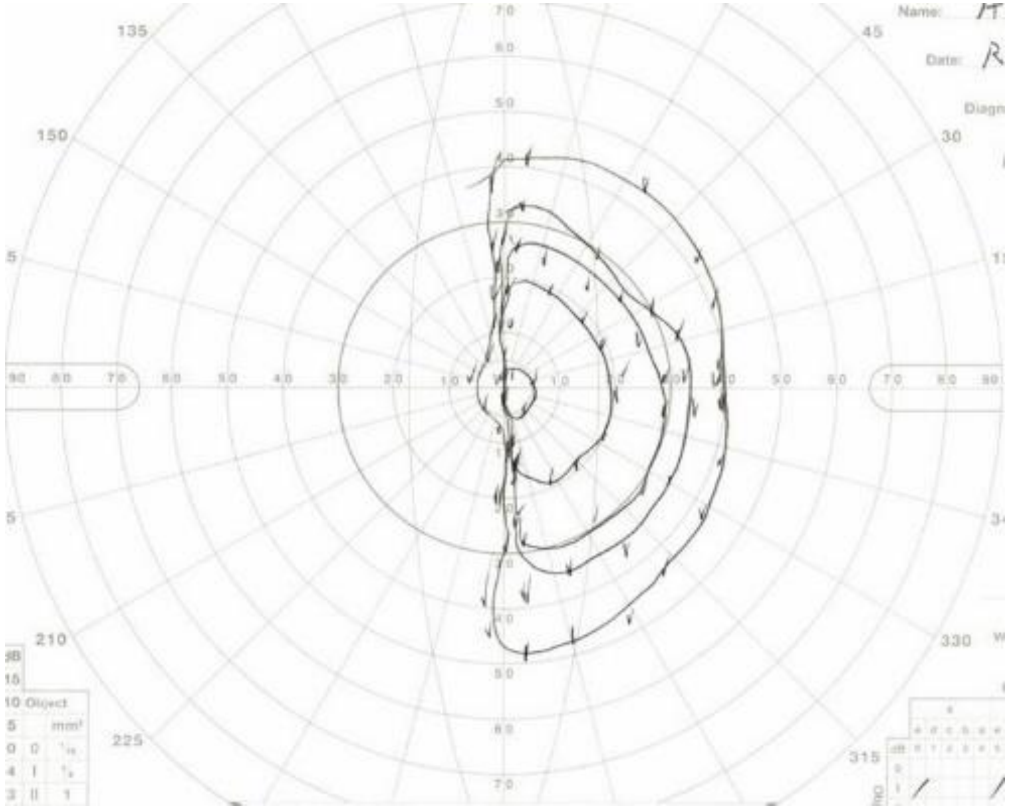

Right eye field

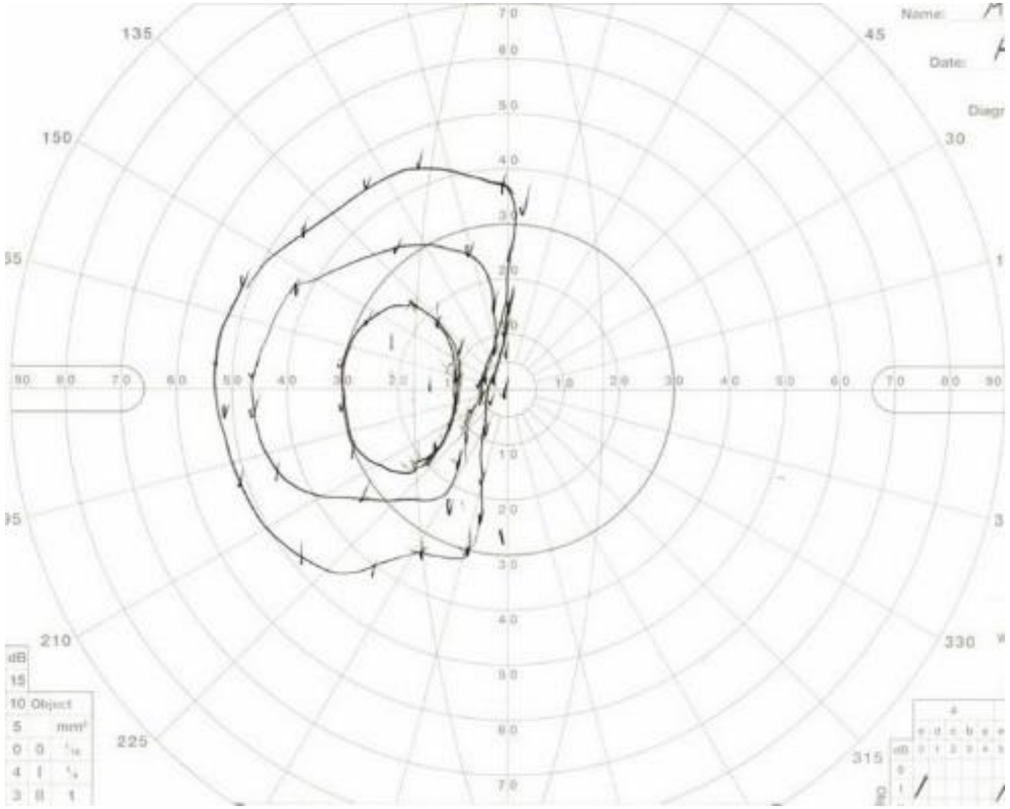

Visual acuity: left: 0.04, right: 0.03

Suppl. Figure 2.

Brain MRI with Gd

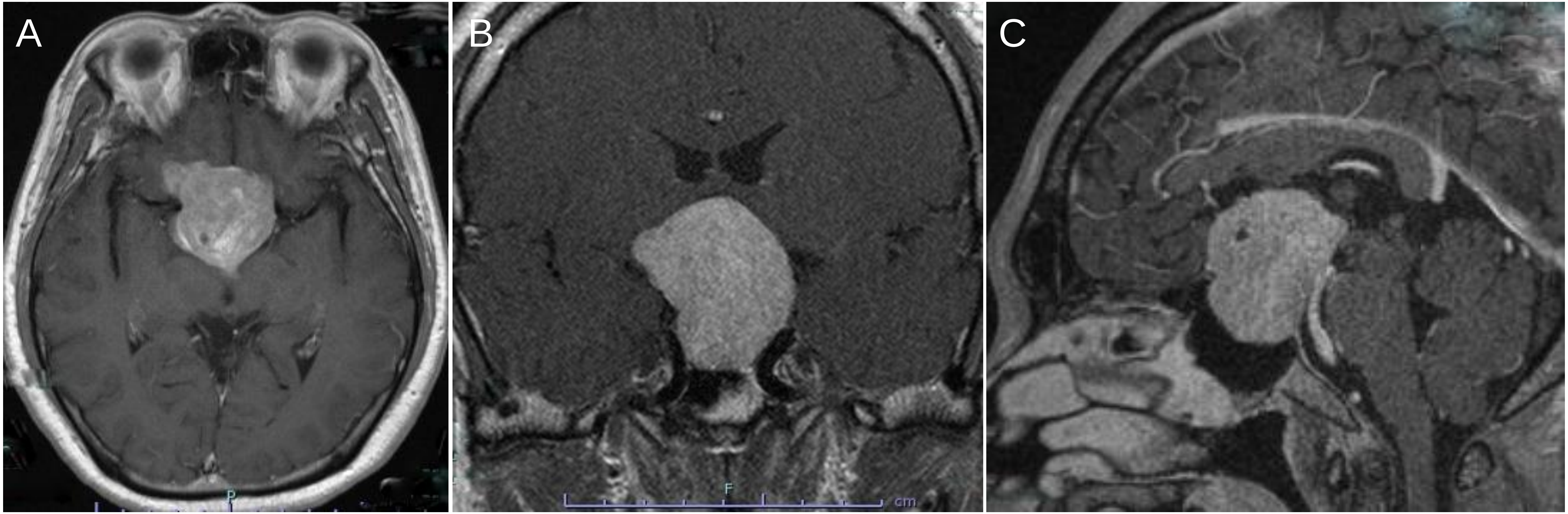

Suppl. Table 1

Parameter	Laboratory data	Normal range
TSH, μIU/mL	3.28	0.50-5.00
FT3, ng/dL	0.78 (↓)	0.90-1.70
FT4, pg/mL	2.5	2.3-4.0
Cortisol, μg/dL	0.8 (↓)	7.07-19.60
ACTH, pg/mL	15.1	7.2-63.3
LH, mIU/mL	4.51	1.71-8.59
FSH, mIU/mL	17.40 (↑)	1.49-12.40
PRL, ng/mL	15.0 (↑)	4.3-13.7
Testosterone, ng/mL	128.0 (↓)	131-871
GH, ng/mL	<0.03 (↓)	0.00-2.47
IGF-1, ng/mL	27 (↓)	91-253
AVP, pg/mL	5.6	0.3-4.2

Suppl. Table 2

Parameter	Laboratory data	Normal range
WBC / µL	59×10^2	$33\text{-}86 \times 10^2$
RBC / µL	469×10^4	$435\text{-}555 \times 10^4$
Hb g/dL	13.8	13.7-16.8
Ht %	39.3	40.7-50.1
Plate / µL	17.5×10^4	$15.8\text{-}34.8 \times 10^4$
PT, sec	13.9(↑)	9.8-12.1
APTT, sec	32	24-39
D-dymer µg/mL	0.8	<1.0

Suppl. Figure 3.

Left eye field

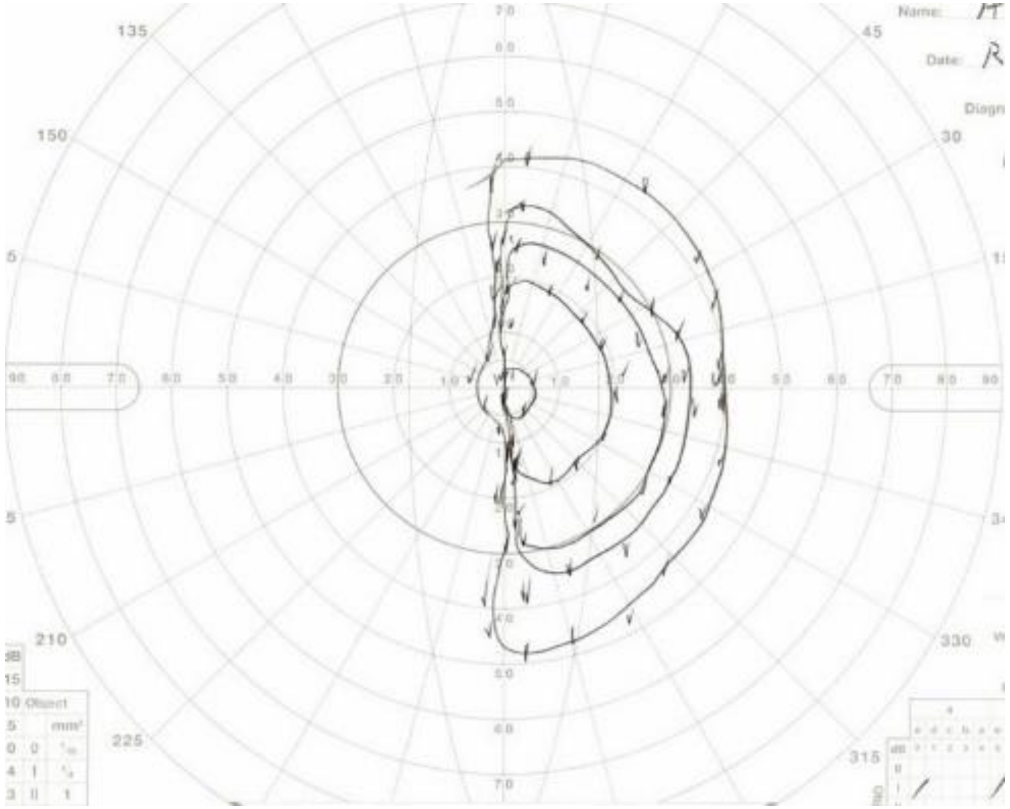

Right eye field

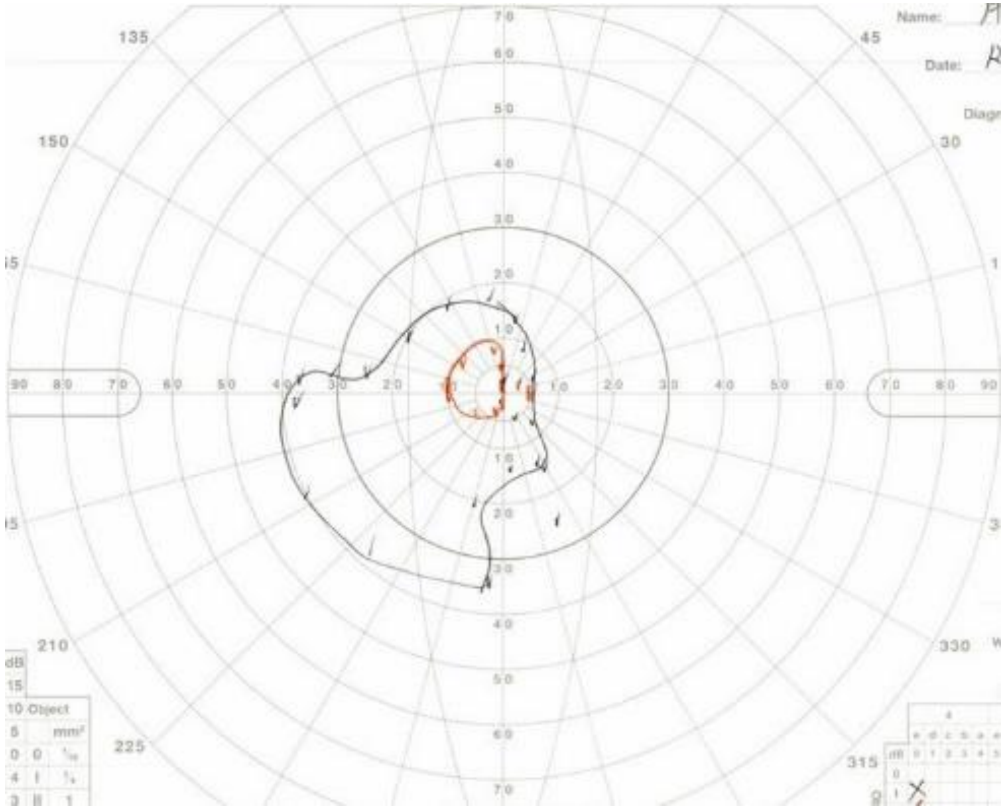

Visual acuity: left: 0.7, right: 0.01

Supple. Figure 4.

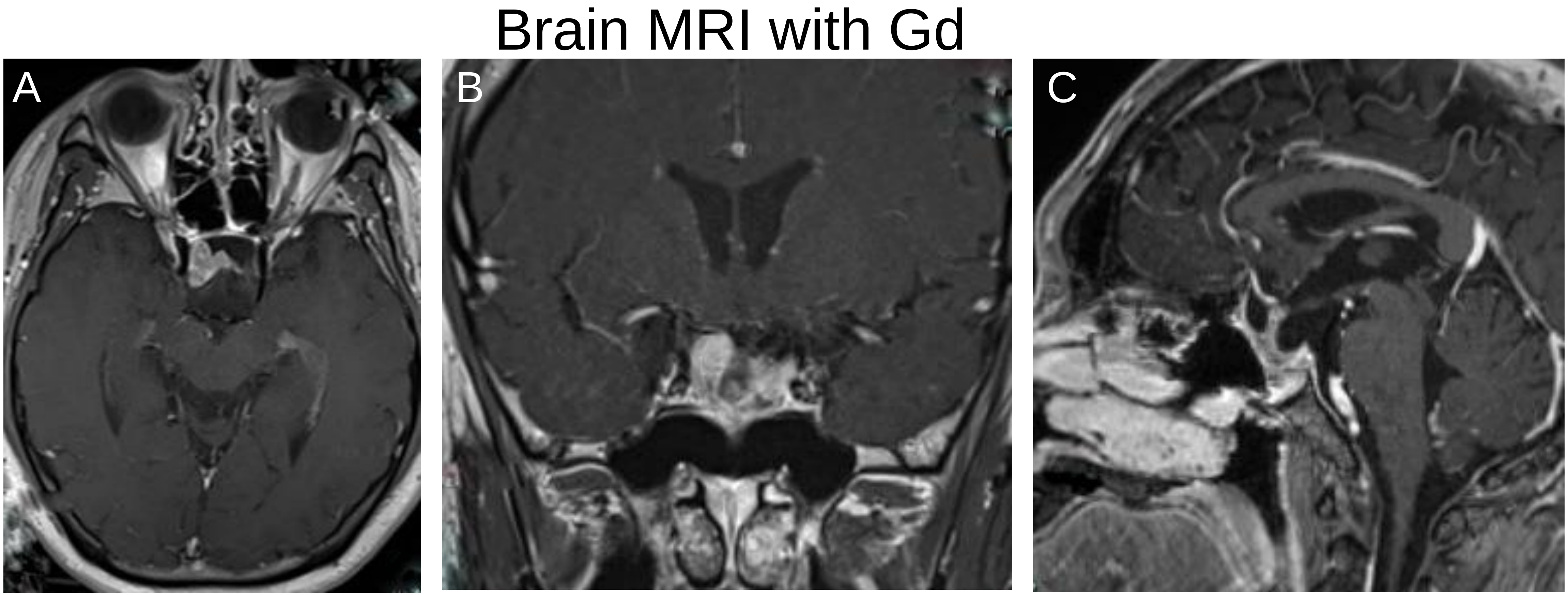

Supplement: Supplementary file 1 [file Datasheet1.pdf]
